# A Review of Bioactive Compounds in Oyster Shell and Tissues

**DOI:** 10.3389/fbioe.2022.913839

**Published:** 2022-06-06

**Authors:** Selvakumari Ulagesan, Sathish Krishnan, Taek-Jeong Nam, Youn-Hee Choi

**Affiliations:** ^1^ Division of Fisheries Life Sciences, Pukyong National University, Busan, South Korea; ^2^ School of Earth, Ocean, and Atmospheric Sciences, Goa University, Taleigao, India; ^3^ Institute of Fisheries Sciences, Pukyong National University, Busan, South Korea

**Keywords:** marine organisms, oyster, bioactive peptides, protein hydrolysates, oyster shell, the biocidal activity of oyster, oyster peptide

## Abstract

Oysters are saltwater bivalves with high nutritional and medicinal value that are consumed widely around the world. As well as being highly nutritious, oysters are a low-calorie, low-cholesterol source of protein and an exceptional source of zinc, which strengthens the immune system; and a rich source of bioactive compounds, which comprise various biological activities. The present review summarizes the biological applications and bioactive compounds from oyster shells, whole tissue, gill tissue, and mantle tissue. The various biological compounds present in an oyster shell, and their chemical constituents, have applications in the food, pharmaceutical, and medical industries. Bioactive peptides and proteins obtained from the whole, mantle, and gill tissues of oysters exhibit antioxidant, antimicrobial, antihypertensive, anticancer, antifatigue, anticoagulant, and anti-wrinkle effects, as well as enhance osteoblast differentiation. This review clearly shows that oysters have great potential for functional food production and that various compounds therein can have pharmaceutical applications.

## Introduction

Oyster is an important and extensively farmed marine resource with high commercial value (Cheng et al., 2021; Hao et al., 2021). Oyster aquaculture has been practiced for over 2,000 years (Campbell and Hall, 2018). According to the Food and Agriculture Organization (FAO, 2018), global oyster production stands at around 6.1 million tons per year, and worldwide, oyster exports increased threefold from 1997 to 2017. Oysters are highly nutritious and possess medicinal value. Oysters have high protein, active polysaccharides, taurine, vitamin, and mineral contents, and are also low in fat (Guo et al., 2020). Many researchers aim to extract bioactive materials from various oyster parts for biomedical applications.

More than 100 species of oyster are cultured worldwide, including Suminoe oyster (*Crassostrea ariakensis*), Zhe oyster (*Crassostrea plicatula*), and Pacific cupped oyster (*Crassostrea gigas*) (Botta et al., 2020). These oysters are excellent sources of nutrition. The protein, glycogen, and fat contents of oyster flesh (dry flesh weight) are approximately 39.1–53.1%, 21.6–38%, and 7.8–8.7%, respectively (Linehan et al., 1999). Thus, oysters are an excellent source of protein and provide vital nutrients with a wide range of bioactive effects (Guo et al., 2020).

**FIGURE 1:**
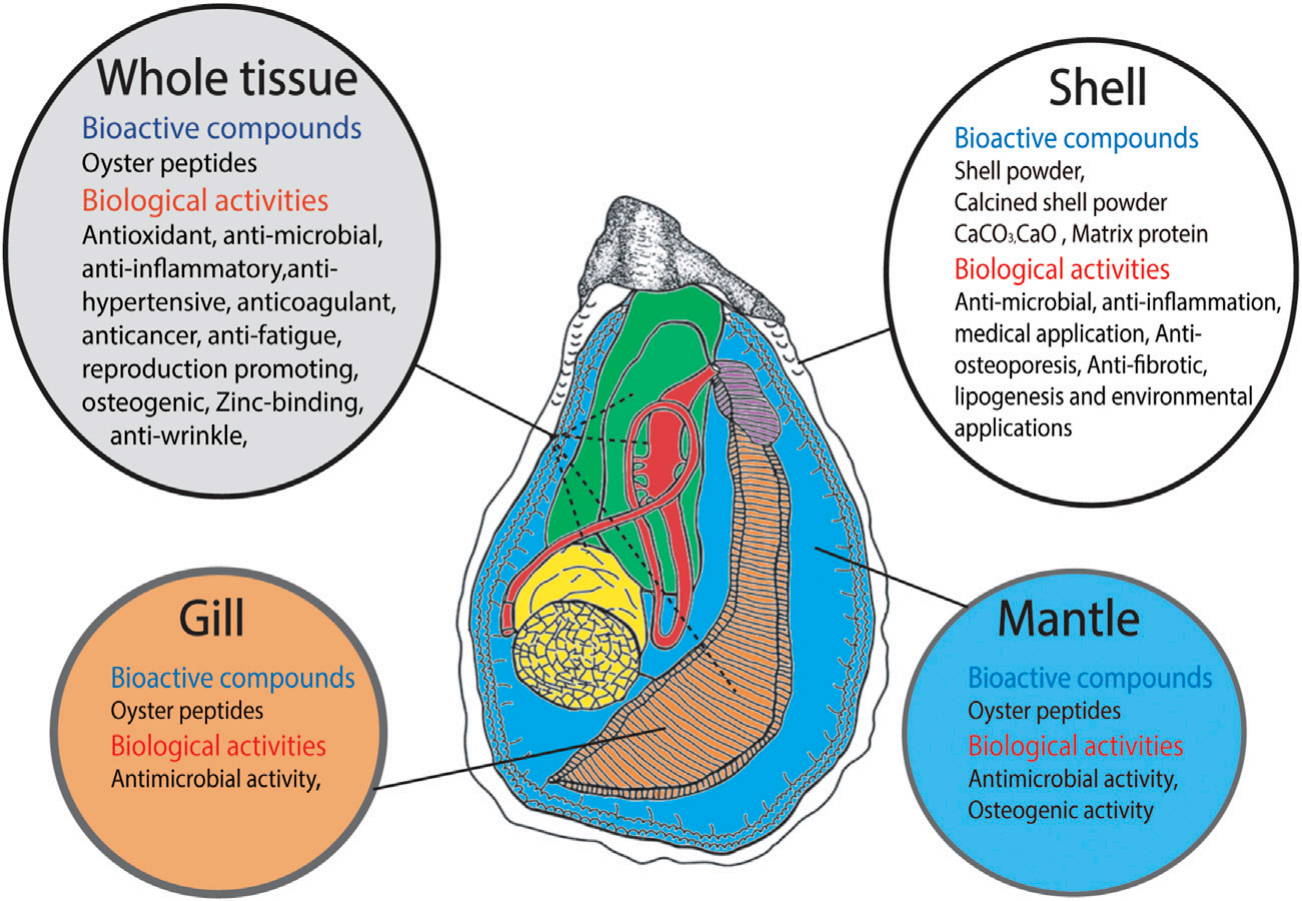
Schematic representation of Bioactive compounds derived from different parts of oyster such as shell, whole tissue, gill tissue and mantle tissue and their biological activities.

Oyster protein can be defragmented into a large number of peptides with high bioactivity. Recently, oyster protein hydrolysates (OPHs) and peptides have attracted attention due to their stability and diverse biological activities (Xie et al., 2018; Guo et al., 2020). Many studies have described the bioactive properties of oyster peptides (OPs), including antioxidant, antitumor, immunomodulatory, antimicrobial, antiviral, antihypertensive, anti-inflammatory, antifungal, anticancer, antimelanogenic, anti-wrinkle, anti-fatigue, anticoagulant, antithrombotic, and osteogenic effects. OPs may also enhance spatial learning and memory, acetylcholinesterase activity, and sexual function, as well as serve as angiotensin-converting enzyme (ACE) inhibitors (Wang et al., 2020 and references therein, Zhang et al., 2021 and references therein, Hao et al., 2021 and references therein). Oyster protein can reduce blood pressure (Achour et al., 1997; Tanaka et al., 2006). Moreover, some studies have reported bioactive effects of compounds in oyster shell, including anti-inflammatory, antiosteoporosis, antifibrotic, antimicrobial, and antifungal effects, and lipogenesis inhibition (Lee et al., 2013; Xing et al., 2013; Han et al., 2015; Latire et al., 2017; Tran et al., 2015; Feng et al., 2021; Figure 1).

Oysters are rich in selenium, which supports a number of cellular functions including heavy metal detoxification. Additionally, oyster soft tissue has a higher zinc (Zn) content than most seafoods (Li et al., 2019). Zn-chelating peptides extracted from oysters have attracted wide attention. Chen et al. (2013) demonstrated that (Figure 1) the peptide HLRQEEKEEVTVGSLK, produced *via* oyster protein hydrolysis, has the ability to bind Zhang et al., 2018 demonstrated that the OPH-Zn complex enhances Zn bioaccessibility.

This review aims to provide insight into the bioactive compounds found in whole, gill, and mantle tissues of oysters, as well as the shell. We first focus on the extensive bioactivity of OPHs, peptides, and matrix proteins in an oyster shell, shell extracts, and calcified shell powder, then discuss future directions for studies on bioactive compounds in oysters.

## Oyster Shell

**FIGURE 2:**
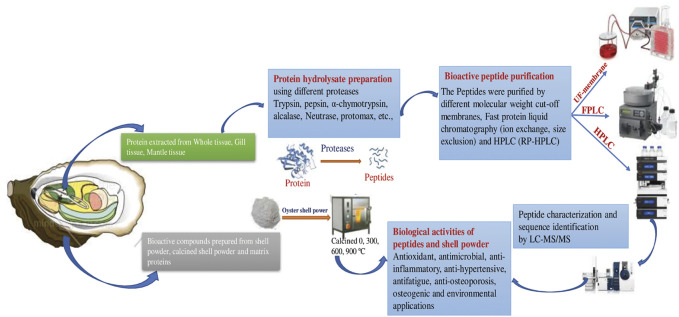
Schematic representation of Preparation and applications of Bioactive peptides from oyster tissue and shell.

Oysters are economically and ecologically important shellfish known for their delicious meat and calcareous shell. The oyster shell comprises an organic matrix and minerals that protect soft tissue (Upadhyay et al., 2016). The shell accounts for about 60% of the total oyster weight (Xing et al., 2013). Oyster shell disposal is associated with waste accumulation and water and marine pollution, due to improper landfill and microbial activity, as well as off-odor issues due to the use of cheap disposal methods, high management costs, and negative effects on soil pH caused by inappropriate recycling (e.g., use as fertilizer). However, oyster waste products have potential as safe and ecofriendly functional compounds. The use of these waste products as biocompatible antimicrobials could improve human health and waste management (Sadeghi et al., 2019). Oyster shells mainly consist of calcium carbonate (CaCO_3_; ∼95%) in addition to a small proportion of organic matrix proteins (∼0.1–5%), which are also called skeleton/shell proteins (Upadhyay et al., 2016; Figure 2).

### Antimicrobial and Biocidal Activities

Calcium-based compounds such as calcium oxide (CaO) and calcium hydroxide [Ca(OH)_2_] can be derived from oyster shell. CaO is extensively used as a catalyst in industrial research and tissue engineering. Calcined oyster shell powder has attracted considerable attention due to its biocompatibility and antimicrobial and biocidal activities. Hence, calcined oyster shell could serve as a good antimicrobial alternative in food processing and food packaging. Adding these natural antimicrobial additives to processed and fresh foods could be a safe means of ensuring food quality. The antimicrobial activity of oyster shell relies primarily on the alkalinity of CaO, which is a major compound in calcined oyster shell that increases the surrounding pH. Calcium ions derived from CaO react with cardiolipin (a major lipid in the bacterial cell membrane), leading to cell wall rupture and the generation of reactive oxygen species (ROS) and free radicals, which strongly affect cell integrity. The antifungal activities of CaO are also related to its alkalinity and ROS generation (Sadeghi et al., 2019).

Over the last 2 decades, many studies have revealed antimicrobial and antifungal activities of oyster shell powder presented in (Table 1). Oikawa et al. (2000) reported that calcined shell powder derived from various species (i.e., oyster, scallop, and clam) exerted strong antimicrobial activity by retarding aerobic bacterial growth and suppressing *Escherichia coli*. Choi et al. (2006) observed that 0.05% oyster shell powder significantly improved the quality of kimchi during storage by reducing and maintaining the numbers of aerobic and lactic bacteria, respectively. Similarly, Kim et al. (2007) reported that the shelf life of tofu could be extended by up to 2 days by adding 0.05–0.2% oyster shell powder, which reduced microbial expression to maintain quality and freshness. Jung et al. (2010) reported that oyster shell powder prolonged the storage time (to 80 days at 5°C) and quality of gat kimchi by reducing lactic acid bacteria, yeast, and *E. coli* compared to the control. A study on the antifungal activity of calcined oyster shells, with heat-treated (1,050°C) CaO as the major compound, demonstrated strong biocidal activity against *Physalospora piricola* and *Rhizoctonia solani*. That study also reported antifungal activity from non-calcined oyster shell, which could be due to the alkalinity of CaCO_3_ in the slurry phase (Xing et al., 2013).

**TABLE 1:** Bioactive compounds derived from oyster shell and their biological applications.

Species	Application	Bioactive compound	Results	References
Oyster	Antimicrobial activity	Calcined shell powder	Suppressing the growth of *Escherichia coli*	Oikawa et al. (2000)
Oyster		0.05% of Shell powder	Improving the quality of kimchi by reducing the growth of aerobic bacteria and increasing lactic bacteria	Choi et al. (2006)
Oyster		0.05–0.2% of shell powder	Reducing he micobial number and increase the self life of tofu	Kim et al. (2007)
Oyster		Shell powder additives	Reducing the microbial number and prolonged the and quality of Gat kimchi	Jung et al. (2010)
Oyster		Heat trated calcined oyster shell (CaO) and non-calined oyster shell	Calcined shellpowder showed biocidal activity against the *Physalospora piricola* and *Rhizoctonia solani* and non-calcine shell powder showed antifungal activity	Xing et al. (2013)
Oyster		Calcined shell powder	Inhibited the microbial growth and increased the self life of pork ham	Choi et al. (2014)
Oyster		Calcined shell powder	Inhibited the food borne disease microorganisms (*Staphylococcus aureus*),	Chen et al. (2015)
			*Listeria monocytogenes*, *Salmonella typhimurium*, *Enterobacter aerogenes*, and *Proteus vulgaris*)	
Oyster		Propylene film with calcined shell powder	Enhanced antimicrobial efficiency without cytotoxicity	Tsou et al. (2019)
Oyster	Anti-inflammatory activity	Oyster shell extract	Suppressed the NO production, decreased the expression of the oyster iNOS, COX-2 and NF-κB and inhibites the production of IL-1β, IL-6, and TNF-α in LPS stimulated Raw 264.7 cells	Lee et al. (2013)
Oyster	Bone tissue bioengineering	Nacre as bone graft sustituion	Narce binds directely with newly formed bone without any fiborous tissue formation	Atlan et al. (1999)
Oyster		Molecules of nacre	Activates the osteogenesis in bone marrow cells	Lamghari et al. (1999)
*Crassostrea gigas*		Water soluble matrix protein (WSMP)	Identified protein with osteogenic activity which are responsible for the bone remodelling and biocompatibility	Oliveira et al. (2012)
Oyster		Nacre powder	osteogenic differentiation of human bone marrow mesenchymal stem cells	Flausse et al. (2013)
Oyster		Nacre particle and its soluble protein matrix	Induce the differentation in human bone cells (osteoinductive capacity)	Green et al. (2015)
*Crassostrea gigas*	Anti-osteooresis activity	WSMP	Promote the osteogenesis and inhibits osteoclast absorption	Feng et al. (2021)
Oyster	Anti-fibrotic activity	Shell extract	Enhances the catabolic pathway of human dermal fibroblasts	
Oyster	Lipogenesis inhibition	Oyster shell extract	lipid-lowering effect *via* inhibition of lipogenesis and diminished the cellular triglyceride level	Khoi et al., 2015
Oyster	Environmental application	Oyster shell	Used to remove pollutants, organics, neurotoxin and nitrogen	Bonnard. 2021
Oyster	Environmental application	Oyster shell powder	Mitigate the harmful algal bloom by reducing phosphate, nitrogen and COD	Huh et al. (2016)

Choi et al. (2014) increased the shelf life of ham by adding calcined shell powder, which inhibited microbial growth. Similarly, Chen et al. (2015) derived an antimicrobial agent through the calcination of oyster, hard clam, and sea urchin shells that suppressed the growth of foodborne microorganisms such as *Staphylococcus aureus*, *Listeria monocytogenes*, *Salmonella typhimurium*, *Enterobacter aerogenes*, and *Proteus vulgaris*. However, non-calcined oyster shell powder did not inhibit microbial growth. The addition of antimicrobial agents to polymer matrices enhances shelf life and prevents foodborne diseases. Tsou et al. (2019) observed that the addition of calcined oyster shells to propylene film enhanced antimicrobial efficiency without causing any cytotoxicity. Thus, calcined oyster shell powder is a good option for increasing the shelf life of food products and preventing foodborne diseases.

Lee et al. (2013) showed that oyster shell extract can significantly suppress the production of nitric oxide (NO) and decrease the expression of inducible nitric oxide synthase (iNOS), cyclooxygenase-2 (COX-2), and nuclear factor (NF)-κB. Additionally, oyster shell extract significantly inhibited the production of interleukin (IL)-1β, IL-6, and tumor necrosis factor (TNF)-α in lipopolysaccharide (LPS)-stimulated RAW264.7 cells, thus exerting an anti-inflammatory effect.

### Medical Applications

Along with high CaCO_3_ content, the organic matrix network plays a vital role in biomineral formationin oyster shells. It comprises macromolecules such as polysaccharides (mostly chitin), water soluble and insoluble proteins (including glycol proteins), and lipids, in addition to smaller molecules such as pigments, free amino acids, and short peptides (Marin et al., 2012). These biomolecules are distinguished based on extraction methods and solubility, with matrix types including the water-soluble matrix (WSM), ethanol-soluble matrix (ESM), acid-soluble matrix (ASM), acid-insoluble matrix (AIM), ethylenediaminetetraacetic acid (EDTA)-soluble matrix (EDTASM), EDTA-insoluble matrix (EDTAIM), and fat-soluble matrix (FSM; (Bonnard, 2021). In addition to regulating biomineralization, the WSM also promotes nacre biological activities such as cell recruitment, differentiation, and stimulation. Due to its beneficial biological activities, nacre (and its biomatrix) is used in traditional pharmaceutical preparations to stimulate bone growth and enhance bone density (Chaturvedi et al., 2013 and references therein). Recent *in vitro* and *in vivo* studies also suggest that nacre is a biocompatible and biodegradable material with osteoinductive, osteointegrative, and osteoconductive properties. Thus, nacre (and its biomatrix) has been evaluated as a potential bone substitute. In fact, nacre has been applied in bone tissue bioengineering as early as 1931 (in the jawbone of a Mayan individual; Green et al., 2015).

A major breakthrough in bone graft substitution using nacre was achieved by Lopez et al. (1992). Atlan et al. (1999) subsequently investigated the interface between bone and nacre in sheep, showing that nacre binds directly to newly formed bone without the need for intervening fibrous tissue. *In vitro* and *in vivo* studies have demonstrated that nacre contains molecules capable of activating osteogenic bone marrow cells (Lamghari et al., 1999). Using proteomics, proteins with osteogenic activity were identified among WSM proteins (WSMPs) from *C. gigas* nacre. These proteins are important for bone remodeling and biocompatibility (Oliveira et al., 2012). The effects of alginate hydrogels, including nacre powder, on osteogenic differentiation of human bone marrow mesenchymal stem cells have also been investigated (Flausse et al., 2013).

Chaturvedi et al. (2013) showed that water-soluble bioactive compounds in nacre possess antioxidant activity and promote osteoblast differentiation. A study on the osteogenic potency of human mesenchymal stem cells indicated that nacre and its soluble protein matrix induce early differentiation of human bone cells *via* osteoinductive effects (Green et al., 2015). Studies have also been conducted on nacre powder mixed with blood, nacre chips, nacre prostheses, and nacre matrix proteins, as well as the effects of nacre WSM on alkaline phosphate activity in MRC-5 fibroblasts and osteogenic activities of nacre WSM and ESM (for a review, see Zhang et al., 2017). Recent *in vitro* and *in vivo* studies demonstrated anti-osteoporosis effects of WSMPs from *C. gigas* that result from the promotion of osteogenesis and inhibition of osteoclast absorption (Feng et al., 2021). Shell extracts also activate the catabolic pathway of human dermal fibroblasts and are thus used in anti-fibrotic strategies, especially in the scleroderma. Latire et al. (2017) and Khoi et al. (2015) investigated the ability of oyster shell extract to inhibit lipogenesis and thus provide a lipid-lowering effect (especially of cellular triglycerides). These studies suggest that oyster shell extracts such as those from the WSM and ESM and WSMPs have a wide range of therapeutic and medicinal effects, including anti-osteoporotic, anti-fibrotic, and osteogenic activities as well as lipogenesis inhibition. Thus, they can play a role in human tissue engineering.

### Environmental and Other Apllications of Shell Waste/Byproducts

Oysters reduce the eutrophication of water bodies and may reduce the amounts of metal cations, plastic particles, and other chemicals in water. Oyster shells can be used to remove pollutants such as certain anions (phosphate [PO_4_^3-^], F^−^, and NO_3_^−^) and cations (Cu, Ni, Mn, As, U, Th, Pb, Fe, Zn, and Co), antibiotics, neurotoxins, and excess nitrogen (N) (Bonnard, 2021). Oyster shell powder has also been used to improve the water quality of lakes by facilitating the removal of algal blooms. This was achieved by reducing total PO_4_^3-^ (by 97%), N (by 91%), and chemical oxygen demand (by 51%; Huh et al., 2016). Moreover, oyster shell powder has been used to prevent the migration of N from sediment (to improve the sediment-water quality) and to suppress eutrophication and control harmful algal blooms in the marine environment (Khirul et al., 2020). Shell waste is also used as a soil conditioner and acts as a pH buffer, sorbent or fertilizer. The hydrogen carbonate content of the oyster shell made it the best neutralizer of acidic soil. Treatment of acidic soil with oyster shells neutralizes the soil as well as increases the Ca^2+^, Mg^2+^, K^+^ and Na^+^ and the stabilization of heavy metals with low solubility that is less exchangeable upon lixiviation (Du et al., 2011; Moon et al., 2014). Calcinated oyster shells are also effective in removing air pollutants such as SO2, SO3, H2S, and NO2 in dry or wet processes as well as CO2 sequestration. Combination of oyster shell and Polyvinyl chloride (PVC), neutralizes harmful hydrochloric acid which results from PVC incineration mimicking the activity of commercially available calcium carbonate and with CaCl2 as by-products. Oyster shell wastes are also used in material synthesis (Filler incomposite, foaming agent, template, support for catalysts, source of sodium, calcium, and, calcium carbonate) and used as building materials (Limestone and aggregate) and cosmetic ingredients (Bonnard, 2021).

## Oyster Tissues

Oyster meat accounts for about half of the dry weight of an oyster (Linehan et al., 1999). Various enzymes have been used to digest oyster meat, and bioactive peptides have been purified from OPHs. OPs possess a wide range of bioactivities that vary according to the receptors involved. This section deals with the various bioactive effects of OPs, including antioxidant, anti-inflammatory, anticancer, antimicrobial, antihypertensive, anticoagulant, antithrombotic, and antifatigue effects; they also inhibit ACE (Figure 2).

### Antioxidants

Antioxidants are free-radical scavengers that prevent cell damage by disrupting the radical chain reaction underlying lipid peroxidation (by scavenging ROS, which play a major role in many diseases) (Kim et al., 2007; Papachristoforou et al., 2020). During normal metabolic reactions, free radicals are produced in cells and tissues, adversely affecting biomolecules such as nucleic acids, lipids, and proteins. They also affect redox status and promote oxidative stress. Free radical-associated oxidative stress is involved with diabetes mellitus, neurodegenerative disorders (e.g., Parkinson’s disease, Alzheimer’s disease, and multiple sclerosis), cardiovascular diseases (e.g., atherosclerosis and hypertension), respiratory diseases, pulmonary dysfunction, cataracts, rheumatoid arthritis, and various cancers (Phaniendra et al., 2015). Some natural antioxidants, such as ascorbic acid, tocopherol, and catechin, are common in foods, medicines, and pharmaceuticals; accordingly, they are used in industries in which the application of synthetic antioxidants (such as butylated hydroxytoluene, tert-butyl hydroquinone, and propyl gallate) is restricted due to the latter’s toxic effects.

Oyster peptides exhibit significant antioxidant activity, and several bioactive peptides have been purified from oyster tissue *via* enzymatic degradation. Presented in Table 2. DNA is the ultimate target of ROS-related oxidative damage. The antioxidative peptide LKQELEDLLEKQE, isolated from the gastrointestinal digestive system of *C. gigas*, scavenged cellular and hydroxyl radicals (Qian et al., 2008). OPs were isolated from *Crassostrea talienwhannensis* meat *via* the action of digestive proteases including papain, neutrase, and alcalase. Hydroxylates derived *via* alcalase digestion exhibited high antioxidant activity (Dong et al., 2010). A study on OPs isolated from *Ostrea plicatula* meat using neutral proteinase reported scavenging effects against hydroxyl radicals (e.g., 1,1-diphenyl-2-picrylhydrazyl) and superoxide anion radicals (Hao et al., 2013). In a similar study, the tissue of *Saccostrea cucullata* was digested using protease enzyme, and seven peptides were obtained (SCAP 1–7). These OPHs exhibited potential for donating hydrogen atoms and scavenging hydrogen peroxide, hydroxyl, and diphenyl-picrylhydrazyl (DPPH) radicals. Among SCAP 1–7, SCAP 1, 3, and 7 exhibited the highest scavenging ability for DPPH radicals (Umayaparvathi et al., 2014). Wang et al. (2014) isolated two OPs (PVMGA and QHGV) from the meat of *C. talienwhanensis* with high antioxidative activities, as reflected in their hydroxyl and DPPH radical scavenging activities. *Crassostrea gigas* meat can be digested by food-grade enzymes such as alcalase, bromelin, and neutral proteases. Among OPs, those derived from hydrolysates exhibited the strongest scavenging activity against DPPH and hydroxyl radicals (Zhang et al., 2015). OPs from enzymatic hydrolysates of *Crassostrea madrasensis* exhibited excellent antioxidant activity and inhibited lipid peroxidation (Asha et al., 2016). Interestingly, OPs extracted from *Ostrea rivularis* by proteases exhibited oxygen radical absorbance capacity (ORAC) and cellular antioxidant activity in a HepG2 cell model (Miao et al., 2018). In a similar study, the antioxidant peptide YA, extracted from oysters, exhibited dose-dependent DPPH and 3-ethylbenzothiazoline-6-sulfonic acid (ABTS) radical scavenging activity (Xie et al., 2018). Moreover, Zhang et al. (2019) reported higher antioxidant activity in alcalase-hydrolyzed oyster meat (*Crassostrea rivularis*) compared to meat not subjected to gastrointestinal digestion. *In vivo*, alcalase-hydrolyzed oyster meat contained high levels of antioxidant enzymes such as glutathione peroxidase, superoxide dismutase, and hydrogen peroxidase, which increased and decreased the levels of T-AOC and malonaldehyde in mice, respectively (Zhang et al., 2019). In a recent study, oyster (*C. talienwhanensis*) protein was hydrolyzed with trypsin under various conditions to obtain peptides in an optimized manner using response surface methodology (Wang et al., 2020). Hydrophobic OP fractions (PEP-1, PEP-2, TRYP-2, MIX-2, and TRYP-2) were extracted from *C. talienwhanensis* and exhibited high antioxidant and DPPH scavenging activities *in vitro*, according to *in vitro* ferric reducing ability of plasma and ORAC assays.

**TABLE 2:** Antioxidant peptides derived from oyster whole tissue.

Species	Enzyme used	Oyster peptides and/or MW	Results	References
*Crassostrea gigas*	Pepsin, trypsin, α-chymotrypsin	LKQELEDLLEKQE (1600 Da)	Scavenged cellular radicals and protective effect on hydroxyl radicals generated DNA damage	Qian et al. (2008)
*Crassostrea talienwhannensis*	papain, neutrase, and alcalase	(<1 kDa)	Alcalase derived hydrolysates showed the strongest overall antioxidant activity	Dong et al. (2010)
*Ostrea plicatula Gmelin*	neutral proteinase		scavenging effects against the hydroxyl radicals	Hao et al. (2013)
			DPPH, and superoxide anion radicals	
*Saccostrea cucullata*	Protease	SCAP 1–7	Peptides (SCAP 1, 3, and 7) had the highest scavenging ability on DPPH radicals	Umayaparvathi et al. (2014)
*Crassostrea talienwhanensis*	Subtilisin	PVMGA (518 Da) and QHGV	DPPH and hydroxyl radical scavenging activity	Wang et al. (2014)
*Crassostrea gigas*	Food grade proteases, alcalase, bromelin, and neutral protease		Alcalase derived hydrolysates showed the maximum scavenging asainst DPPH and hydroxyl radicles	Zhang et al. (2015)
*Crassostrea madrasensis*	Papain	ISIGGQPAGRIVM (1297.72 Da)	DPPH and hydroxyl radical-scavenging activity,	Asha et al. (2016)
			Ferric reducing and iron chelating activity and lipid peroxidation inhibition	
*Ostrea rivularis*	Protease	(<6 kDa)	ABTS and DPPH radical-scavenging activity oxygen radical absorbance capacity (ORAC) and cellular antioxidant activity (CAA) in a HepG2 cells	Miao et al. (2018)
oyster	Protamex, Neutrase	YA	Dose dependent DPPH and ABTS radical scavenging activity	Xie et al. (2018)
	Microbial transglutaminase			
*Crassostrea rivularis*	*Crassostrea rivularis*	Alcalase	*In-vivo* study showed the increased activity of antioxidant enzymes (GSH-Px, SOD and CAT)	Zhang et al. (2019)
		and the T-AOC levels and reduced MDA level		
*Crassostrea talienwhanensis*	Trypsin	PVMGA (518 Da), QHGV (440 Da)	DPPH and hydroxyl radical-scavenging activity	Wang et al. (2020)
*Crassostrea talienwhanensis*	Pepsin, trypsin, Maxipro PSP	PEP-1, PEP-2, TRYP-2, and MIX-2	DPPH and hydroxyl radical-scavenging activity	Qian et al. (2020)

### Antimicrobial Activity

Antimicrobial peptides (AMPs) constitute a small class of naturally abundant peptides that play a vital role in the innate immunity of various organisms. The discovery of antibiotic-resistant microorganisms prompted the development of synthetic AMPs with applications in medicine, the food industry, animal husbandry, and aquaculture. Many studies have demonstrated that AMPs have inhibitory effects against bacteria, fungi, parasites, and viruses (Huan et al., 2020). AMPs consist of amino acids with a positive net charge that form an α helix or β sheet. Based on their structure and components, AMPs promote pore formation and disrupt the negatively charged bacterial membrane *via* ionic and hydrophobic interactions. Hundreds of AMPs have been isolated from various organisms, from fungi to humans, and are major components of the innate immune defense of many marine invertebrates including crustaceans, chelicerates, and urochordates (Hao et al., 2021).

Oyster blood cells, gills, and mantle tissue contain abundant AMPs. AMPs from oysters affect Gram-positive (*Bacillus subtilis*, *Listeria monocytogenes*, *Staphylococcus aureus*, etc.) and Gram-negative bacteria (*E. coli, Vibrio parahaemolyticus*, and *Vibrio harveyi*) as well as fungi (*Fusarium oxysporum*, *Botrytis cinerea*, and *Penicillium expansum*) presented in Table 3. Some studies have shown that the oyster protein peptides LLEYSI and LLEYSL inhibit HIV-1 protease, which is a crucial enzyme for viral maturation and a major target of HIV-1 treatment (Bednasz et al., 2019). These peptides showed superiority over pepstatin A as HIV-1 protease inhibitors (Lee and Maruyama 1998). Vero cells are model organisms for studying the antiviral activities of oyster AMPs. Previous studies have demonstrated cytotoxic effects of AMPs on Vero cells carrying pseudorabies virus and herpes simplex virus type 1 (Olicard et al., 2005; Zeng et al., 2008). OPs are classified as AMPs, antimicrobial proteins (>50 kDa), and ubiquitous proteins, and exhibit antimicrobial activity among other functions. Liu et al. (2007) reported that CgPep33, a novel and cysteine-rich AMP isolated from enzymatic hydrolysates of Pacific oyster (*C. gigas*), exhibited high inhibitory activity against the fungus *Botrytis cinerea*. A similar study by Liu et al. (2008) showed that CgPep33 exhibited activity against Gram-positive and -negative bacteria and fungi. Cysteine and aromatic residues were also posited to play a crucial role in CgPep33 antimicrobial activity. Interestingly, chemically synthesized AMPs with short amino-acid chains, referred to as Cg-Prp (found in *C. gigas*), exhibited antimicrobial activity against Gram-positive and -negative bacteria and fungi (Gueguen et al., 2006). Rosa et al. (2011) identified so-called “big defensin form” AMP sequences involved in genomic organization and the regulation of gene expression. CgMolluscidin is an oyster dibasic residue repeat-rich AMP comprising 23 basic (lysine) and 15 hydrophobic amino acids that exhibits activity against Gram-positive and -negative bacteria. The amino-acid sequence cgTβ, isolated from *C. gigas*, is similar to β-thymosin in terms of its antibacterial activity. Moreover, protein hydrolysates from digestion by food-grade proteases were shown to exhibit antimicrobial activity against human pathogenic bacteria (Nam et al., 2015; Zhang et al., 2015).

**TABLE 3:** Antimicrobial peptide derived from oyster whole Tissue.

Species	Name of AMP	Enzyme used	Oyster peptides and/or MW	Results	References
*Crassostrea gigas*		Thermolysin	LLEYSI, LLEYSL	Inhibit the HIV-1 protease	Lee & Maruyama (1998)
*Crassostrea virginica*		Plasma peptides	(<10 kDa)	Against the Gram-positive bacteria (*B. megaterium*)	Anderson and Beaven (2001)
*Crassostrea gigas*		SPE		Cytotoxic activity against vero cells	Olicard et al. (2005)
*Crassostrea gigas*	Cg-Defh1 and Cg-Defh2		GFGCPRDQYKCNSHCQSIGCRAGYCDAVTLWLRCTCTDCNGKK and GFGCPGDQYECNRHCRSIGCRAGYCDAVTLWLRCTCTGCSGKK	Recombinant AMP’s based on prokaryotic expression of *Escherichia coli*	Gonzalez et al. (2007)
*Crassostrea gigas*	CgPep33	Alcalase		Antifungul activity against *Botrytis cinerea*	Liu et al. (2007)
*Crassostrea gigas*	CgPep33	Alcalase		Antibacterial activity against Gram-positive bacteria (*Bacillus subtilis*, *Streptococcus aureus*) and Gram-negative bacteria (*Escherichia coli*, *Pseudomonas aeruginosa*), fungi (*B. cinerea*, *Penicillium expansum*)	Liu et al. (2008)
*Crassostrea gigas*	Cg-Prp	Alcalase	ILENLLARSTNEDREGSIFDTGPIRRPKPRPRPRPEG	Against Gram-positive bacteria (*Micrococcus lysodeikticus*, Brevibacterium stationis, Microbacterium maritypicum), Gram-negative bacteria (*E. coli* SBS363, *Enterobacter cloacae*, *Erwinia carotovora*, *Klebsiella pneumonia*), fungi (*Fusarium oxysporum, Botrytis cinerea*)	Gueguen et al. (2009)
*Crassostrea gigas*	Cg-BigDef1		QAQALLPIASYAGLTVSAPVFAALVTVYGAYALYRYNIRRRENSYQRIRSDHDSHSCANNRGWCRPTCFSHEYTDWFNNDVCGSYRCCRPGRSG (10.7 kDa)	Against gram positive and negative bacteria	Rosa et al. (2011)
	Cg-BigDef1		QAQALLPIASYAGLAVSPPVFAALVTAYGVYALYRYNIRRENSDHDSHSCANNRGWCRPTCYSYEYTDWFNNDVCGSYRCCRPGRRG		
	Cg-BigDef1		QAQILLPIASYAGLTVTAPVFAALVAAYGIYAVTRYAIRKRRIVMYSDSHSCANNRGWCRESCFSHEYTDWANTFGVCGSYFCCRPY		
*Crassostrea gigas*	cgTβ	Trypsin	(4656.4 Da)	Against Gram-positive bacteria (*B. subtilis* KCTC	Nam et al. (2015)
				1021) and Gram-negative bacteria (*E. coli* D31)	
*Crassostrea gigas*		Alcalase, bromelin, neutrase	(6500 Da)	Against Gram-positive bacteria (*Listeria monocytogenes*, *Staphylococcus aureus*) and Gram-negative bacteria (*E. coli*, *Pseudomonas aeruginosa*, *Vibriyo parahaemolytius*, *V. harveyi*)	Zhang et al. (2015)

### Antihypertensive Activity

Hypertension is reaching epidemic proportions and currently affects 15–20% of adults worldwide. Hypertension is a severe chronic health issue that increases the risk of arteriosclerosis, stroke, myocardial infarction, and end-stage renal disease. ACE is a Zn-metallopeptidase that plays a crucial role in regulating blood pressure. ACE catalyzes angiotensin conversion from an inactive decapeptide (angiotensin I) into a potent vasoconstrictor octapeptide (angiotensin II) and inactivates the antihypertensive vasodilator bradykinin (Wang et al., 2008). High angiotensin II levels and low NO production elevate the risk of high blood pressure (Oparil et al., 2018). Many studies have been carried out on the synthesis of ACE inhibitors, such as captopril, enalapril, alacepril, and lisinopril, which are used in the treatment of hypertension and heart failure. However, side effects led to the search for ACE inhibitors from other sources, including milk, hemoglobin, fish, buckwheat, beef, soybean, and fermented foods (Wang et al., 2008). Rich sources of ACE-inhibitory peptides include squid and jellyfish, among other invertebrates. Similarly, ACE-inhibitory peptides derived from oysters exert significant antihypertensive effects. Oyster extracts containing low-molecular-weight Ops were also found to inhibit ACE and reduce systolic blood pressure in spontaneously hypertensive rats (Hao et al., 2021 and references therein).

A peptide sequence (VVYPWTQRF) purified from protein hydrolysates of the oyster *C. talienwhanensis* was observed to exert antihypertensive activity (Wang et al., 2008). An *in vivo* study demonstrated competitive inhibition of spontaneously hypertensive rats by peptides (592.9 Da) isolated from fermented oyster sauce, as well as a reduction in blood pressure in the rats (Je et al., 2005). Two novel peptides (HLHT and GWA) isolated from the meat of pearl oyster exhibited high ACE-inhibitory activity, and protein hydrolysates exerted a strong antihypertensive effect in SRH rats. These results indicate that OPs could serve as ingredients of functional foods for hypertension (Liu et al., 2019). *In silico* and *in vitro* studies of protein hydrolysates produced from the pepsins bromelain and papain, isolated from the Portuguese oyster *Crassostrea angulata*, revealed high DPP-IV activity in pepsin hydrolysates. Bioactivity assays also indicated higher activity of low-molecular-weight pepsin hydrolysate compared to crude hydrolysate (Gomez et al., 2019). Another interesting study showed that heat treatment of *C. gigas* yielded more OPs with ACE-inhibiting effects. In fact, that study considered oyster protein to be the best natural source of ACE-inhibitory peptides, such that those peptides could be incorporated into functional foods for hypertension (Guo et al., 2020).

### Anti-Inflammatory Activity

Inflammation is generally caused by the release of chemicals from tissues and migrating cells, and serves as defense against noxious stimuli and microbial infections. Inflammation promotes rapid tissue repair but also plays a role in diseases such as rheumatoid arthritis, chronic bronchitis, asthma, and cancer (Hwang et al., 2019). Bacterial endotoxins present in the cell wall of bacteria, such as LPS, activate the mitogen-activated protein kinase (MAPK) pathway, downstream NF-κB, and cyclic AMP-responsive element. This leads to macrophage proliferation and upregulates inducible NO and COX-2 expression (Choi et al., 2009). COX-2 plays a major role in converting arachidonic acid into prostaglandins, which regulate immune functions. iNOS is regulated by cytokines and transcriptional activation of macrophage cells (Hwang et al., 2019); elevated levels of iNOS cause NO accumulation and promote the production of cytokines (TNF-α and ILs), among other inflammatory factors (Mollace et al., 2005).

Recent studies have shown that OPs exert robust anti-inflammatory effects by suppressing proinflammatory cytokines. For example, a purified peptide sequence (Gln-Cys-Gln-Cys-Ala-Val-Glu-Gly-Gly-Leu at the N-terminal position) from *C. gigas* exhibits strong anti-inflammatory activity. RAW264.7 cell lines are generally used in studies on anti-inflammatory effects with LPS. In one study on protein hydrolysates of *C. gigas*, an NO assay with RAW264.7 cells was performed (Hwang et al., 2012). YA, a multifunctional oyster-derived peptide, exhibited anti-inflammatory activity (Xie et al., 2018). Oyster-derived β-thymosin, a ubiquitous, low-molecular-weight polypeptide, inhibited NO production in LPS-induced RAW264.7 cells to the same extent as human β-thymosin. Oyster β-thymosin also inhibited the expression of inflammatory cytokines, such as TNF-α, IL-1β, and IL-6, and suppressed the nuclear translocation of phosphorylated NF-κB and the degradation of inhibitory κB in LPS-induced RAW264.7 cells (Hwang et al., 2019). Qian et al. (2020) demonstrated anti-inflammatory effects of four peptides (PEP-1, PEP-2, TRYP-2, and MIX-2) isolated from oyster soft tissue, based on downregulation of TNF-α and mRNA expression of proinflammatory mediators (IL-1β, IL-6, and iNOS) in LPS-stimulated RAW264.7 cells.

### Anticoagulant Activity

Cardiovascular diseases (CVDs) are the biggest threat to human health. CVDs include deep vein thrombosis, pulmonary embolism, stroke, and ischemic heart disease. Thrombosis and hypercoagulability are the primary drivers of CVD. Thrombosis can present as primary or secondary hemostasis; in the latter condition, soluble fibrinogen is converted into insoluble fibrin by thrombin, a serine protease and major component of the coagulation cascade. Hence, thrombin is a major target for anticoagulant agents (Cheng et al., 2021). Recent studies have demonstrated anticoagulant effects of OPs. For instance, the antithrombotic peptides DFEEIPEEYLQ and LSKEEIEEAKEV were isolated from oysters, with the former exhibiting thrombin-inhibiting activity including prolongation of partial thromboplastin and thrombin times. Meanwhile, LSKEEIEEAKEV interacts with thrombin (*via* carbon-hydrogen and conventional hydrogen bonds) through a salt bridge involving various amino acids (Cheng et al., 2018; Chen et al., 2019). Other OPs, such as TARNEANVNIY and P-3-CG, also exhibit anticoagulant activity. P-3-CG inhibits the binding of fibrinogen and thrombin, thus limiting coagulation (Cheng et al., 2018; Cheng et al., 2021).

### Anticancer Activity

Multiple compounds from marine organisms, including bioactive peptides, exhibit antioxidant properties. Recent studies have demonstrated that bioactive peptides from marine organisms, such as fish and mussels, inhibit cancer cell growth while being of low toxicity. Moreover, peptides from tuna dark muscle and sepia ink exhibited cytotoxicity against human breast and prostate cancer cells, respectively (Hao et al., 2021 and references therein). Similarly, OPs have exhibited anticancer activity, including preventing the proliferation of cancer cells. An *in vivo* study of oligopeptide-enriched hydrolysates from *C. gigas* reported inhibition of the growth of sarcoma S-180 in BALB/c mice in a dose-dependent manner (Wang et al., 2010). Peña-Ramos et al., 2004 reported anticancer activities of OPs in relation to hydrophobic amino acids, such as leucine, isoleucine, methionine, and tryptophan. Moreover, Cheong et al. (2013) revealed that the OP HFNIGNRCLC causes apoptosis of prostate, breast, and lung cancer cells but not normal liver cells. The OP SCAP1-LANAK, isolated from *Saccostrea cucullata*, exhibited anticancer activity against human colon carcinoma cell lines (Umayaparvathi et al., 2014).

### Other Potential Biological Activities

Oyster protein and peptides exhibited various biological activities which is presented in Table 4. Fatigue can prevent an individual from performing their usual activities of daily living and can also be the initial symptom of Parkinson’s disease (Kluger et al., 2016). *In vivo* studies showed that mice treated with OPs exhibited strong swimming performance, high endurance, and elevated levels of glycogen in the muscle and liver during vigorous exercise (Miao et al., 2018). Lactic acid and blood urea levels, as indicators of anti-fatigue effects and endurance in humans, are regulated by OPs (Hao et al., 2013; Xiao et al., 2020). OPs additionally mitigate skin damage caused by oxidative stress, inflammation, and collagen degradation. The ability of OPs to potentiate the anti-wrinkle effects of human fibroblasts has been well-studied (Kim et al., 2015; Bang and Choung, 2019; Peng et al., 2020).

**TABLE 4:** Bioactive peptides derived from oyster whole tissue.

Biological activity	Species	Enzyme used	Oyster peptides and/or MW	Results	References
Antihypertensive activity	*Crassostrea talienwhanensis*	Pepsin	VVYPWTQRF (1195 Da)	ACE inhibitory activity and decrease the systolic blood pressure (SBP)	Wang et al. (2008)
	Crosse				
	*Crassostrea gigas* (FOS)		(592.9 Da)	ACE-inhibitory activity and decrease the SBP by 12 mmHg after 3 h	Je et al. (2005)
	*Crassostrea gigas*	Trypsin	DLTDY	ACE inhibitory activity and decrease the SBP	Shiozaki et al. (2010)
	*Pinctada fucata*		HLHT and GWA	ACE inhibitory activity and efficient antihypertensive effect on SRH rats	Liu et al. (2019)
	*Crassostrea angulata*	Pepsin, bromelain, and papain		Higher ACE and DPP-IV activity observed in pepsin hydrolysates	Gomez et al. (2019)
Anti-inflammatory activity	*Crassostrea gigas*	Protamex	QCQCAVEGGL	Inhibit NO production in RAW264.7 cells and Decrease and increase the serum IgE and spleen CD4+/CD8+ levels in Male BALB/c mice, respectively	Hwang et al. (2012)
		Microbial transglutaminase, protamex and neutrase	YA	Inhibit NO production in RAW264.7 cells	Xie et al. (2018)
	*Crassostrea gigas*	Trypsin	β-thymosin (4656.4 Da)	Inhibits NO production in LPS-induced RAW264.7 cells and PGE2, iNOS and COX-2 expression; Inhibits the inflammatorycytokines (TNF-α, IL-1β, IL-6), repress the nucleartranslocationof phosphorylated nuclear factor-κB and degradation of inhibitor of nuclear factor-κB	Hwang et al. (2019)
	*Crassostrea talienwhanensis*	Pepsin		Repress inflammatory cytokines and mediators (TNF-α, IL-1β, IL-6, iNOS) at transcription level in LPS-induced RAW264.7 cells	Qian et al. (2020)
Anticoagulant activity	*Crassostrea gigas*	Pepsin, trypsin papain and nuetral protease	DFEEIPEEYLQ and TARNEANVNIY (1264.36)	Thrombin inhibition including prolonged the activated partial thromboplastin	Cheng et al. (2018)
	*Crassostrea gigas*	Pepsin and pancreatin	LSKEEIEEAKEV	Prolong the APTT and TT, inhibit thrombin	Chen et al. (2018)
	*Crassostrea gigas*	Pepsin	P-3-CG	Prolong the APTT and decrease the mortality in male Kumming mice	Cheng et al. (2021)
Anticancer activity	*Crassostrea gigas*	Protease	Oligopeptide-enriched hydrolysates (<3 kDa)	Inhibition against the growth of sarcoma-s-180 in BALB/c mice	Wang et al. (2010)
	*Crassostrea gigas*	Flavourzyme	HFNIGNRCLC	Cytotoxicity including apoptosis of prostate, breast, and lung cancer cells but not normal liver cells	Cheong et al. (2013)
	*Saccostrea cucullata*	Protease isolated from *Bacillus cereus* SU12	LANAK	Activity against the HT-29 cell lines	Umayaparvathi et al. (2014)
Anti-fatigue	*Ostrea plicatula*	Nutrease		prolonged swimming time and incresed glycogen in liver and gastrocnemius muscle; decrease the serum lactic acid and BUN male Kumming mice	Hao et al. (2013)
	*Gmelin*				
	*Ostrea rivulari*	Compound protease	(<6 kDa)	Increase in exercise tolerance, liver glycogen and muscle glycogen, decrease the levels of BUN in male Kumming mice	Miao et al. (2018)
		Nutrease		Prolong the swimming time, increase the levels of liver glycogen,	Xiao et al. (2020)
				decrease the levels of lactic acid	
					
Anti-wrinkle	*Crassostrea gigas*	Protamex, Neutrase and AMG		Anti-melanogenic activities *via* downregulation of cAMP signaling in B16F10 cells and decreased the number of active melanocytes and melanin granule in UVB-irradiated mice group	Han et al., 2019
Reproduction enhancing					
		Neutrase, papain, bromelain	(343 Da)	Rats with reproductive dysfunction induced by cyclophosphamide exhibited Increase the level of key regulatory protein expression and androgen in the blood	Li et al. (2020)
	*Crassostrea gigas*	SPE	Cg-GnRH-a (pQNYHFSNGWQPa) and CgGnRH-G (pQNYHFSNGWQPG)	Regulate the neurological and endocrine factors and supports the nutrition and energy demand during reproduction	Bigot et al. (2012)
	*Crassostrea iredalei*			Ethanol extract showed the aphrodisiac property	Ridzwan et al. (2013)
Osteogenic	*Crassostrea gigas*	Pepsin, pancreatin	YRGDVVPK (992.40 Da)	Promote the proliferation of MC3T3-E1 cells	Chen et al. (2019)
	*Crassostrea gigas* (fermented oyster extract)			Osteoclastogenesis by inactivating the NF-κB-mediated NFATc1 and c-Fos signaling pathways and scavenging the ROS generation in RAW 264.7 Cells	
Zinc-binding	*Crassostrea gigas*	Pepsin		Peptide-zinc complex promoted the intestinal absorption of zinc *via* the zinc ion channel and the small peptide transport pathway	Li et al. (2019)

Moreover, protein hydrolysates inhibit procollagen and matrix metalloproteinase-1 production, thereby improving skin health and the appearance of wrinkles (Kim et al., 2015). The OP SSDNNDEAK downregulates genes and proteins associated with the cAMP signaling pathway in B16F10 cells, by reducing melanin production and tyrosinase activity (Han et al., 2019). Recent studies on OPs also revealed that they have collagen-restoring capabilities (i.e., anti-wrinkle properties) (Hao et al., 2021). Further, OPs promote sexual reproduction by increasing the expression of key regulatory proteins and androgens, thereby increasing serum estrogen levels and improving male sexual function (Li et al., 2020). For instance, GnRH-like peptides (Cg-GnRH-a and Cg-GnRH-G) isolated from the visceral ganglion of oysters regulate neurological and endocrine factors and provide nutrition and energy for reproduction (Bigot et al., 2012). Ridzwan et al. (2013) demonstrated the aphrodisiac properties of an ethanol extract of *Crassostrea iredalei* in mice and suggested that it could serve as an alternative therapy to enhance male sexual performance.

A fermented oyster extract inhibited osteoclastogenesis by inactivating the NF-κB-mediated NFATc1 and c-Fos signaling pathways and scavenging ROS in RAW264.7 cells (Jeong et al., 2019). A novel peptide (YRGDVVPK) derived from *C. gigas* protein hydrolysates promoted MC3T3-E1 proliferation, possibly by activating the MAPK signaling pathway (Chen et al., 2019). Zinc is an essential nutrient involved in many enzymatic and metabolic processes in humans. A peptide-Zn complex derived from *C. gigas* promoted intestinal absorption of Zn *via* the Zn ion channel and a so-called “small peptide transport pathway” (Li et al., 2019).

## Bioactive Compounds From Oyster Gill and Mantle Tissues

The oyster gill is a complex ciliated organ with major roles in feeding, respiration, and excretion. Cilia of the gill are involved in the generation of strong water currents that pass through numerous branchial chambers to ensure gas exchange between organs and the surrounding medium, as well as the transport of food particles. OPs isolated from the gill exhibit various biological activities Table 5. Many AMPs and proteins have been detected in oyster gill tissue, including defensin (Seo et al., 2017). Oyster defensin (FGCPWNRYQCHSHCRSIGRLGGYCAGSLRLTCTCYRS) was detected in the gill of the American oyster *Crassostrea virginica* (Seo et al., 2005).

**TABLE 5:** Bioactive peptides derived from oyster gill tissue and mantle tissue.

Biological activity	Species	Enzyme used	Oyster peptides and/or MW	Results	References
Anti-microbial	*Crassostrea virginica* (Gill tissue)		GFGCPWNRYQCHSHCRSIGRLGGYCAGSLRLTCTCYRS (4265.0 Da)	Against Gram-positive bacteria (*Lactococcus lactis subsp. lactis, S. aureus*) and Gram-negative	Seo et al. (2005)
				bacteria (*E. coli* D31, *Vibriyo parahemolyticus*)	
	*Crassostrea gigas*	Trypsin	cgUbiquitin (8471 Da)	Against Gram-positive bacteria (*Streptococcus iniae*), Gram-negative bacteria (*V. parahemolyticus*)	Seo et al. (2013a)
	(Gill tissue)				
			cgMolluscidin, AATAKKGAKKADAPAKPKKATKPKSPKKAA	Against Gram-positive bacteria (*B. subtilis*,	Seo et al. (2013b)
			KKAGAKKGVKRAGKKGAKKTTKAKK (5.5 kDa)	*Micrococcus luteus, S. aureus*) and Gram-negative bacteria (*E. coli, Salmonella enterica,V. parahaemolyticus*)	
			AKSKNHTSHNQNRKQHRNGIHRPKTYRYPSMKGVDPKFL KNLKFSKKHNKNTKK(6484.6 Da)	Against Gram-positive bacteria (*Bacillus subtilis*	Seo et al. (2017)
				KCTC 1021) and Gram-negative bacteria	
				(*Escherichia coli* D31, *Vibrio parahaemolyticus)*	
	*Crassostrea gigas*		Cg-Defm, GFGCPGNQLKCNNHCKSISCRAGYCDAATLWL	Against Gram-positive bacteria (*M. lysodeikticus*,	Gueguen et al. (2006)
	(Mantle tissue)		RCTCTDCNGKK	*S. aureus*, *S. hemeolyticus*, *B. megaterium*,	
				*B. stationis*, *M. maritypicum*)	
Osteogenic	*Pinctada fucata* (*Mantle tissue*)		PFMG4	Homology with C1q protein in different species, mainly in MC3T3-E1 osteoblast cell	Wang et al. (2015)

The antimicrobial polypeptides cgUbiquitin and cgMolluscidin were purified from an acidified extract of Pacific oyster (*C. gigas*) gill tissue and exhibited antimicrobial activity against Gram-positive and -negative bacteria (Seo et al., 2013a and; Seo J.-K. et al., 2013). Seo et al. (2017) additionally reported antimicrobial effects (against Gram-positive and -negative bacteria) of 60S ribosomal protein of *C. gigas.* Oyster mantle tissue promotes the formation of oyster shell and its nacre lining. Gueguen et al. (2006) discovered a novel defensin gene in the mantle tissue of *C. gigas*, referred to as Cg-Def, and found that this defensin exhibited intrinsic activity against Gram-positive bacteria but had no effect on Gram-negative bacteria. The mantle gene *Pinctada fucata* mantle gene 4 is highly expressed in mantle tissue and homologous with C1q protein in various species. It is mainly observed to be expressed in MC3T3-E1 osteoblast cultures, suggesting an ability to enhance osteoblast differentiation (Wang et al., 2015).

## Conclusion and Future Perspectives

Oyster is an abundant marine resource, which contains high protein content. Documentation of bioactive compounds which exhibits various biological activities is an emerging and substitute for artificial drug and food additives. Thus, exploring such compound from the marine edible organism will be biocompatible and less toxic. Oyster shell, considered as a major food waste, comprised of the bioactive compounds with biocidal, bioengineering and biological activities, such usages of these food waste will be beneficial to the environmental and waste management. Oyster proteins obtained from oyster tissue, yields proteins hydrolysates when digested by different proteases, which is consisting of low molecular weight peptides and amino acids and their biological applications are well studied. However structural analysis of oyster peptide is still very limited. Therefore, extensive studies on therapeutic proteins and peptides from oysters and their structural and functional characterization using bioinformatics analysis will be beneficial. This review cumulatively documented numerous isolation and biological activity of oyster peptides such as antioxidant, antimicrobial, anticancer, antihypertensive, anticoagulant etc., Besides, most of the experiments have studied the activity of oyster peptides *in vitro*, but not *in vivo*, which will not provide a reference data for the clinical research of oyster peptides. Hence we suggest that the *in vivo* studies of oyster peptides will be advantageous in clinical trial and drug development.
